# Rare case of penile fracture caused by an injury to the crus penis: Delayed repair using the transperineal approach

**DOI:** 10.1002/iju5.12232

**Published:** 2020-11-17

**Authors:** Junki Harada, Yohei Shida, Suzuna Gono, Masahito Masato, Tsutomu Yuno, Tomoaki Hakariya, Toshiharu Kihara, Kanenori Maeda, Yasuyoshi Miyata, Hideki Sakai

**Affiliations:** ^1^ Department of Urology Nagasaki University Graduate School of Biomedical Sciences Nagasaki Japan; ^2^ Department of Urology, Nephrology and Dermatology Maeda Clinic Nagasaki Japan

**Keywords:** adult, delayed repair, erectile dysfunction, penile fracture, pubic bone

## Abstract

**Introduction:**

Penile fracture is a rare urologic emergency, and its surgical treatment is selected based on the damaged site of the penile corpus cavernosum. Penile fractures at the site of the crus penis are quite rare, and there is controversy regarding the preferred method of surgical repair.

**Case presentation:**

A 25‐year‐old Asian man was injured when rolling over in bed. Magnetic resonance imaging showed a tear in the left crus of the penis with a hematoma. Delayed surgery was successfully performed using the transperineal approach. He did not experience pain, dysuria, or erectile dysfunction postoperatively.

**Conclusion:**

Delayed surgical repair using transperineal approach may be useful for penile fractures associated with penile crus injuries.

**Abbreviations & Acronyms**
MRI: magnetic resonance imaging
PF: penile fracture
T2WI: T2 weighted image

**Keynote message**

PF caused by penile crus injury is a rare urological trauma. Although there is controversy regarding the method and timing of crus repair, delayed repair using the transperineal approach may be a useful therapeutic option.

## Introduction

PF is a rare urological emergency that is mostly caused by sexual intercourse or masturbation.^1^ It has been reported that PF injuries are often proximal parts of the penis.^2^ They are usually repaired by circumcising (degloving) or direct lateral incision approach to the damaged sites of the tunica albuginea of the penis.^3^ Α few reports have associated PF with penile crus damage, and a different surgical approach was selected for each case. We report the case of a patient with PF with a tear to the crus penis that was successfully repaired using the transperineal approach.

## Case presentation

**Fig. 1:**
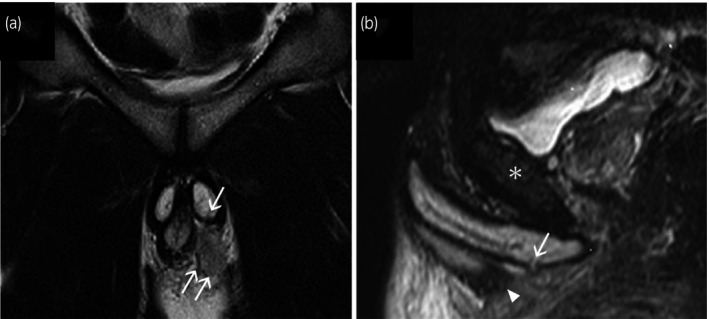
(a) Pelvic MRI coronal T2WI and (b) sagittal T2WI fat suppression. The 6‐mm tear in the tunica albuginea of the left ventral penile crus (single arrow) and a hematoma (double arrows) are presented. Arrowhead points to the bulbospongiosus muscle. The asterisk shows the pubic bone.

**Fig. 2:**
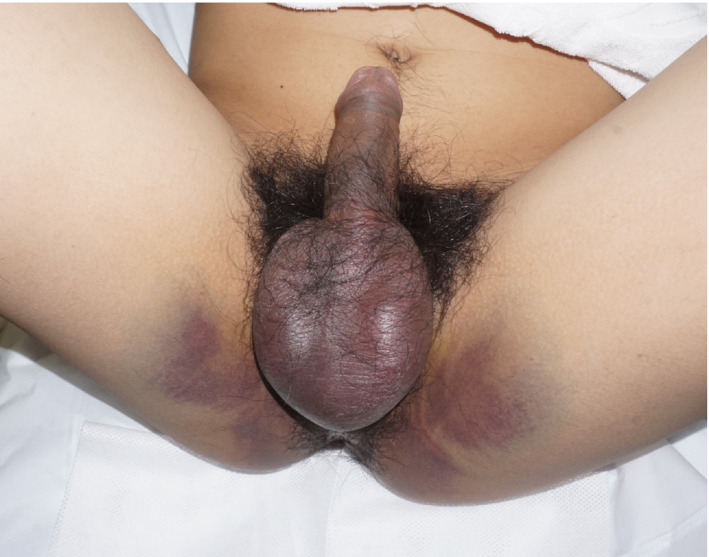
Image obtained during physical examination showing that subcutaneous perineal bleeding has spread to the hip; the penis can also be seen.

**Fig. 3:**
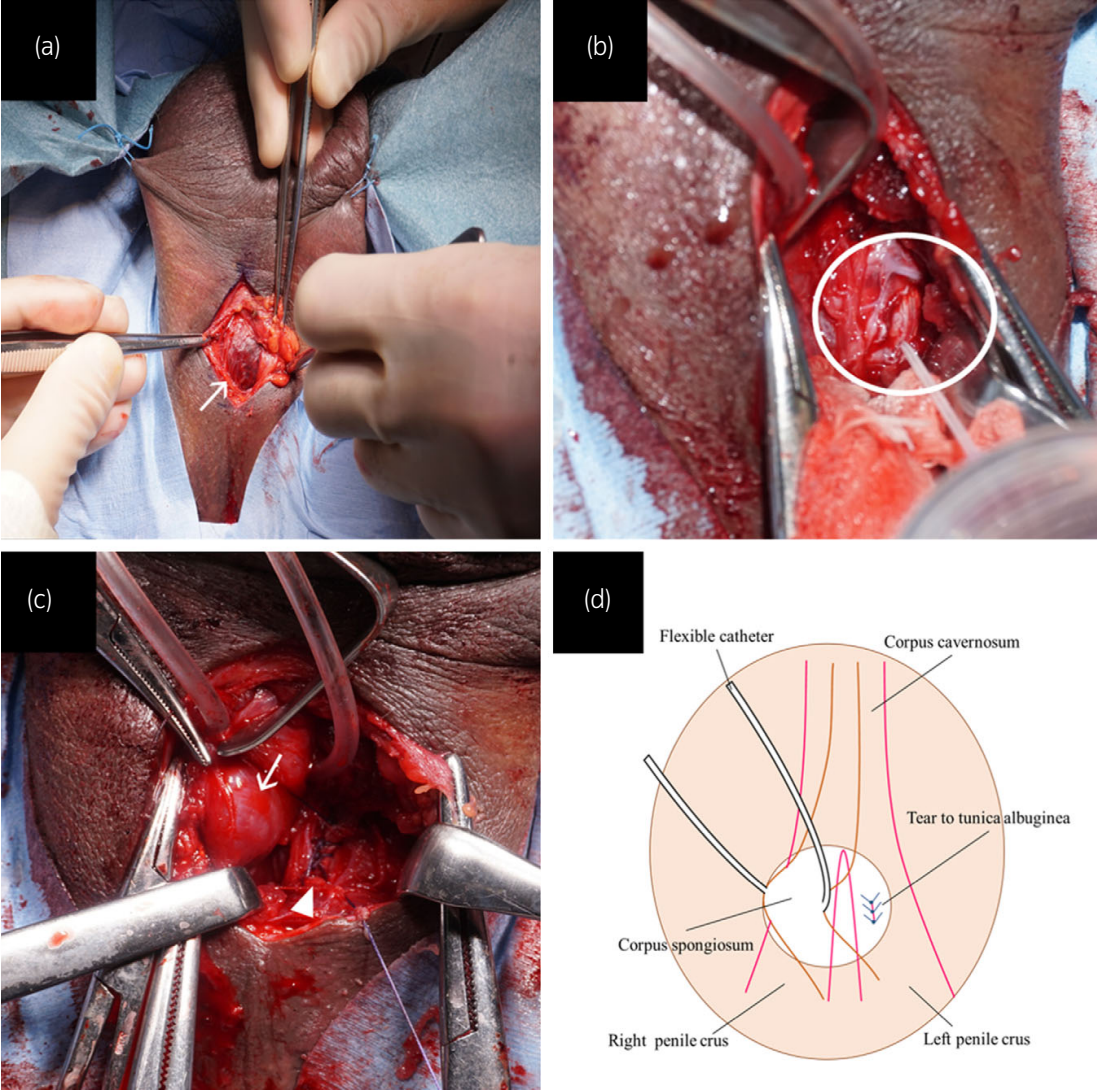
(a) Image obtained intraoperatively, when performing the transperineal approach, showing the bulbospongiosus muscle (arrow). The scrotum is fixed with silk threads. (b) Intraoperative image obtained before repair. The transperineal approach is adopted, and tunica albuginea tear to the left penile crus is visible (circle). (c) Intraoperative image obtained after repair. The corpus spongiosum (arrow) is pulled with a flexible catheter. The tear in the tunica albuginea of the left crus penis is repaired using interrupted sutures (arrowhead). (d) Schematic of the operative field.

A 25‐year‐old Asian man presented to our department 2 days after a penile trauma with perineal pain and erectile dysfunction. The patient was injured during rolling over in bed and heard a “snap” sound just before pain. Physical examination showed swelling of the perineum with subcutaneous bleeding and tenderness. However, the appearance of the penis was normal. His laboratory data were within the normal limits, and hematuria was not detected on urinalysis. Ultrasonography revealed a hematoma in the perineum without any testicular injury. MRI showed a subcutaneous perineal hematoma and a 6‐mm tear to the ventral tunica albuginea of the left crus penis near the bulbospongiosus muscle (Fig. 1). He was diagnosed with PF associated with a penile crus injury. Six days after injury, repair of the tear at the left penile crus was performed. The transperineal approach was selected because the injured area was near the bulbospongiosus muscle. Subcutaneous bleeding expanded into his hip. However, a penis deformity was not found (Fig. 2). Just before surgery, we were able to insert a 14‐Fr Foley’s catheter smoothly through his urethra and no urethral injury was observed. We made a 4‐cm incision in his perineum and identified the bulbospongiosus muscle after removing the hematoma. After dissecting the bulbospongiosus muscle, the corpus spongiosum of the penis was pulled to the right side using an 8‐Fr flexible catheter. The trauma site of the left crus penis was revealed and repaired by interrupted sutures using 3‐0 absorbable sutures (Fig. 3). The day after surgery, Foley’s catheter was removed without complications. The patient had a good recovery and was discharged 6 days after surgery without postoperative complications. At the follow‐up period of approximately 50 days, he did not face perineal pain, dysuria, or erectile dysfunction.

## Discussion

PF associated with penile crus injury is a rare penile trauma, and its mechanisms are uncertain. The tunica albuginea of the corpora cavernosa, one of the toughest fascia in the body, is able to withstand rupture pressures at up to 1500 mmHg. Although the tunica albuginea is 2 mm thick in a flaccid penis, it decreases to approximately 0.25 mm during erection and becomes vulnerable to sudden increases in intracavernosal pressure.^4^ The common PF mechanisms include: acute bending of an erect penis and thrusting against the partner’s perineum or during masturbation.^5^ These mechanisms often lead to rupture of the tunica albuginea of the penile shaft. In contrast, there are only a few cases of penile crus injury, and the mechanisms differ from one case to another.^6^, ^7^ Therefore, it is difficult to determine the precise mechanisms of PF induced by crus injury. In this case, the cause of crus injury might indicate that the left crus was strongly caught between the patient’s pubic bone and bed when he was rolling over during erection. This situation may have led to an increase in intracavernosal pressure of the penile crus and resulted in the tear to the opposite side of the bone. However, we should always consider that it is difficult to identify the true mechanism of PF because many patients feel embarrassed and may not want to disclose the true cause.

The main causes of PF are sexual intercourse (46%), forced flexion (21%), and masturbation (18%), while rolling over in bed is relatively rare (8.2–9.5%).^8^, ^9^ Fracture of the right corpus cavernosum (59.5%) was more common than left‐side fracture (29%) or fracture of both sides (11.5%).^10^ Over 60% of the fractures occurred at the proximal parts of the penis.^3^ In Japan, Ishikawa *et al.* reported that the 20s are the common age for PF, which was often caused by damage to the right corpus (74.1%) in the proximal part of the penis (54.2%).^2^ However, the PF rate associated with sexual intercourse (19.9%) was lower than that in other developed countries.^2^ This may be because Japanese have lesser sexual intercourse than people in other countries.^11^

PF is a urological emergency, which is often repaired immediately. However, a delayed operation is selected in some selected cases.^12^ There is no definitive consensus on the surgical repair timing. Several reports have indicated that the long‐term results of immediate or delayed repair are almost equal. Some authors have recommended delayed repair of PF.^13^, ^14^ It has also been reported that surgical repairs as early as 9 days after PF are useful to preserve penile function.^15^, ^16^ In our case, delayed repair was selected because the patient did not aim for an immediate repair of the penile crus injury due to a temporal relief of his perineal pain.

In two previous cases, penile crus injury was repaired using two different approaches, namely, the transperineal and penoscrotal approaches.^6^, ^7^ The postoperative clinical course was almost good in both cases. However, the latter case required use of phosphodiesterase type 5 inhibitors for approximately 1 month after the surgery.^7^ It is unclear which approach was better or on what basis the two approaches were selected. In this case, we considered it difficult to reach the crus injury using a penoscrotal approach because the injured penile area was near the bulbospongiosus muscle. Therefore, the transperineal approach was selected and led to good outcomes.

## Conclusion

We described a rare case of a patient with PF caused by a penile crus injury. It was managed with delayed repair using the transperineal approach with satisfactory outcomes. The crus injury frequency is quite low, and selection of the method and timing for repair is controversial. Therefore, further studies are needed to determine the appropriate method and timing of surgical repair.

## Conflict of interest

The authors declare no conflict of interest.
